# Prognostic role of computed tomography-based, artificial intelligence-driven waist skeletal muscle volume in uterine endometrial carcinoma

**DOI:** 10.1186/s13244-021-01134-y

**Published:** 2021-12-20

**Authors:** Se Ik Kim, Joo Yeon Chung, Haerin Paik, Aeran Seol, Soon Ho Yoon, Taek Min Kim, Hee Seung Kim, Hyun Hoon Chung, Jeong Yeon Cho, Jae-Weon Kim, Maria Lee

**Affiliations:** 1 Department of Obstetrics and Gynecology, Seoul National University College of Medicine, 101 Daehak-Ro, Jongno-Gu, Seoul, 03080 Republic of Korea; 2 Department of Radiology, Seoul National University College of Medicine, Seoul, 03080 Republic of Korea; 3 Department of Radiology, UMass Memorial Medical Center, Worcester, MA 01605 USA; 4 Department of Obstetrics and Gynecology, Seoul National University Hospital, Seoul, 03080 Republic of Korea

**Keywords:** Endometrial neoplasm, Sarcopenia, Body composition, Prognosis, Artificial intelligence

## Abstract

**Objectives:**

To investigate the impact of computed tomography (CT)-based, artificial intelligence-driven waist skeletal muscle volume on survival outcomes in patients with endometrial cancer.

**Methods:**

We retrospectively identified endometrial cancer patients who received primary surgical treatment between 2014 and 2018 and whose pre-treatment CT scans were available (*n* = 385). Using an artificial intelligence-based tool, the skeletal muscle area (cm^2^) at the third lumbar vertebra (L3) and the skeletal muscle volume (cm^3^) at the waist level were measured. These values were converted to the L3 skeletal muscle index (SMI) and volumetric SMI by normalisation with body height. The relationships between L3, volumetric SMIs, and survival outcomes were evaluated.

**Results:**

Setting 39.0 cm^2^/m^2^ of L3 SMI as cut-off value for sarcopenia, sarcopenia (< 39.0 cm^2^/m^2^, *n* = 177) and non-sarcopenia (≥ 39.0 cm^2^/m^2^, *n* = 208) groups showed similar progression-free survival (PFS; *p* = 0.335) and overall survival (OS; *p* = 0.241). Using the median value, the low-volumetric SMI group (< 206.0 cm^3^/m^3^, *n* = 192) showed significantly worse PFS (3-year survival rate, 77.3% vs. 88.8%; *p* = 0.004) and OS (3-year survival rate, 92.8% vs. 99.4%; *p* = 0.003) than the high-volumetric SMI group (≥ 206.0 cm^3^/m^3^, *n* = 193). In multivariate analyses adjusted for baseline body mass index and other factors, low-volumetric SMI was identified as an independent poor prognostic factor for PFS (adjusted HR, 1.762; 95% CI, 1.051–2.953; *p* = 0.032) and OS (adjusted HR, 5.964; 95% CI, 1.296–27.448; *p* = 0.022).

**Conclusions:**

Waist skeletal muscle volume might be a novel prognostic biomarker in patients with endometrial cancer. Assessing body composition before treatment can provide important prognostic information for such patients.

**Supplementary Information:**

The online version contains supplementary material available at 10.1186/s13244-021-01134-y.

## Key points

- Waist skeletal muscle volume might be a new prognostic biomarker in endometrial cancer.
- Assessment of body composition before treatment can provide prognostic information.
- Volumetric quantification of skeletal muscle appears feasible in patients with endometrial cancer.

## Introduction

Endometrial cancer is a global burden, with 417,367 new cases estimated to occur annually [1]. In the USA, endometrial cancer ranks as the fourth most common female cancer and the sixth leading cause of cancer-related deaths in 2021 [2]. In Korea, the incidence of endometrial cancer has increased progressively, and nowadays, it is the most common gynaecologic malignancy [3, 4]. The Western lifestyle and a significant increase in the incidence of obesity in women caused the rapid increase in the incidence of endometrial cancer and other obesity-related cancers in Korea [4, 5].

Obesity is a well-known risk factor for endometrial cancer, and it is strongly correlated with type 1 endometrial cancer. The risk of endometrial cancer reportedly increased 1.5 times for overweight and over 2.5 times for obese women [6]. Body mass index (BMI) has been widely used as an indicator of excess body fat. In a large cohort study in the USA, a significant trend was observed between higher BMI and increased risk of death from endometrial cancer [7]. Recently, not only excess body fat but also lack of muscle mass, known as sarcopenia, has attracted the attention of researchers for causing adverse survival outcomes in many malignancies, including breast [8, 9], lung [10], and gastric cancers [11].

Studies investigating the prognostic role of pre-treatment sarcopenia in endometrial cancer have shown conflicting results [12–14]. To determine sarcopenia, these studies commonly measured skeletal muscle area from a single computed tomography (CT) scan image, based on previous findings that the third lumbar vertebra (L3)-level cross-sectional image reflects total body muscle mass and adipose tissues well [15, 16]. Beyond the areal measurement, recent technological advances enable the volumetric measurement of a specific body composition component, such as skeletal muscle, visceral fat, and subcutaneous fat, from the CT scans that were not feasible due to the requirement of substantial time and human effort [17]. The volumetric measurement of body composition may contain more abundant and precise information than areal measurements in a single cross-sectional image [18]. Moreover, the artificial intelligence-based tool can process a large amount of imaging data by automatic segmentation and calculation of volumes shorter than a few minutes.

Thus, we aimed to ascertain the impact of CT-based, artificial intelligence-driven waist skeletal muscle volume on survival outcomes in patients with endometrial cancer. Additionally, we investigated the prognostic role of each body composition volume, automatically measured using an artificial intelligence-based tool.

## Materials and methods

### Study population

This single-centre retrospective cohort study was approved by the Institutional Review Board (No. H-2012-027-1178) and performed according to the principles of the Declaration of Helsinki. The requirement for informed consent was waived.

From the institution’s Endometrial Cancer Cohort, we identified patients who met the following criteria: (1) diagnosed with endometrial cancer at the age of 20 years or more; (2) received primary surgical treatment between January 2014 and December 2018; and (3) whose pre-treatment CT scans, obtained less than a month before the primary surgery, were stored in the Picture Archiving and Communication System. Meanwhile, we excluded the patients who (1) were not able to retrieve or did not undergo pre-treatment CT scans; (2) had other active malignancies before and at the time of endometrial cancer diagnosis; (3) received hormone therapy, chemotherapy, or radiation prior to surgical treatment; (4) had insufficient clinicopathologic data; and (5) were lost to follow-up during adjuvant treatment or within 3 months without disease recurrence.

### Data collection

Reviewing medical records and pathologic reports, we collected the patients’ clinicopathologic data, including age, comorbidities, serum CA-125 levels, histologic type and grade, and the 2009 International Federation of Gynaecology and Obstetrics (FIGO) stage. Histological grade 3 tumours were considered high-grade disease. We also collected data on pathologic risk factors, such as myometrial invasion and lymphovascular space invasion (LVSI), and post-operative adjuvant treatment. Based on the pre-treatment BMI, all patients were classified into four categories according to the World Health Organization’s (WHO) criteria for Asian populations [19]: < 18.5 kg/m^2^ (underweight), 18.5–22.9 kg/m^2^ (normal), 23.0–24.9 kg/m^2^ (overweight), and ≥ 25.0 kg/m^2^ (obese).

The patients underwent a physical examination and a blood test for serum cancer antigen 125 (CA-125) levels every 3 to 4 months for the first 2 years, every 6 months for the next 2 years, and annually thereafter. Imaging studies were conducted according to physician preference or when symptoms or examination findings were suspicious for recurrence. Progression-free survival (PFS) and overall survival (OS) were defined as the time interval from the date of surgery to the date of disease progression confirmed by the Response Evaluation Criteria in Solid Tumours version 1.1 [20] and cancer-related death or the end of the study, respectively.

### CT image analysis

For body composition analysis, we uploaded the anonymised digital imaging and communications in medicine images of pre-treatment CT scans to the commercially available artificial intelligence-based software (DEEPCATCH v1.0.0.0; MEDICALIP Co. Ltd., Seoul, Korea). This software automatically executes the following procedures: (1) measurement of L3-level skeletal muscle area (cm^2^); (2) volumetric segmentation of skeletal muscle, abdominal visceral fat, and subcutaneous fat, providing a Dice similarity score of 97%, compared with manual segmentation [17]; (3) labelling the abdominal waist between the top of the iliac crest and the lower border of the rib cage, according to the WHO guidelines for measurement of waist circumference [21]; and (4) quantification of each volumetric segmentation (cm^3^). One radiologist (S.H.Y.), expertise in body composition analysis, confirmed all the procedures and results.

**Fig. 1:**
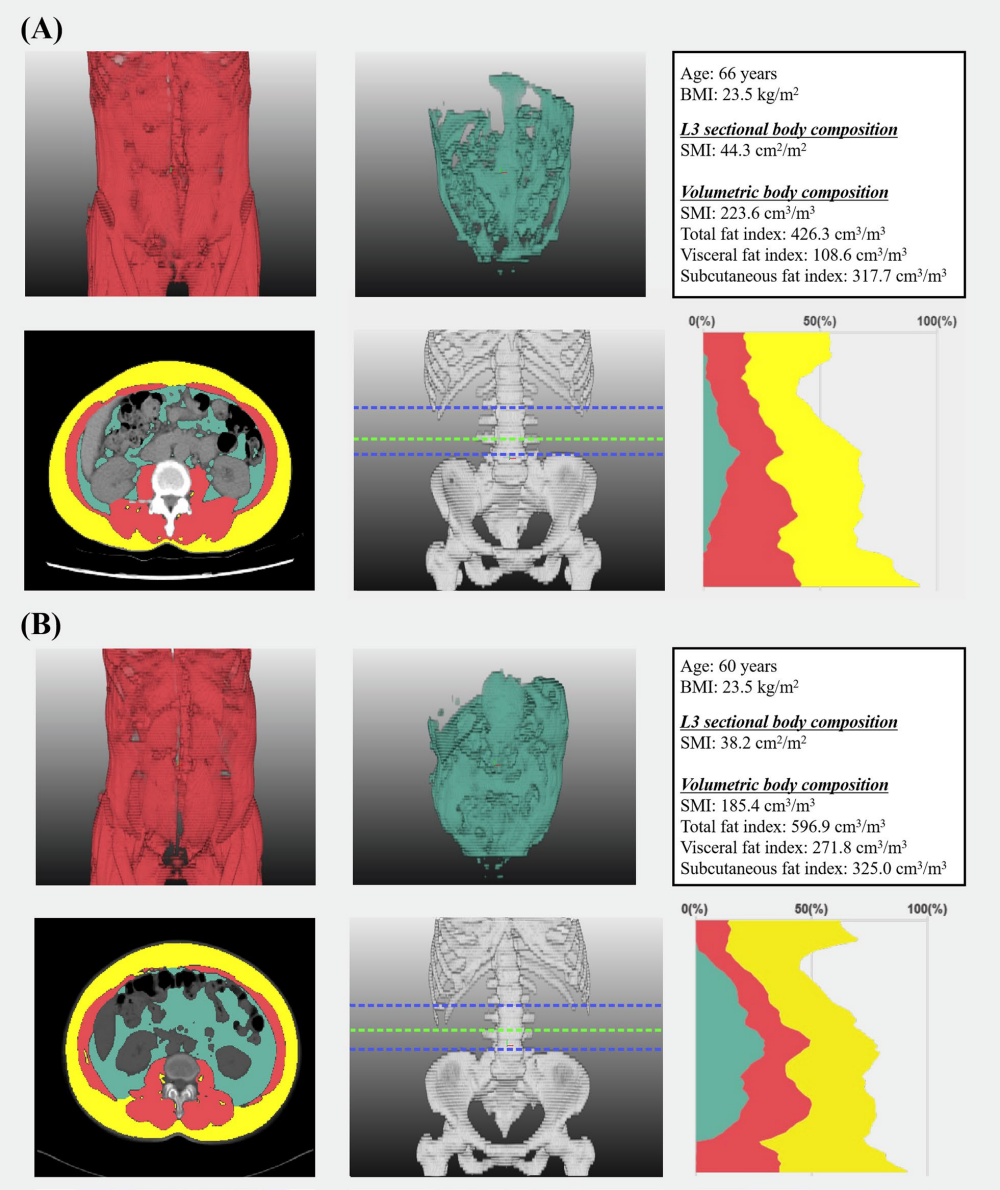
Evaluation of body composition using CT image. Despite similar baseline BMI, two patients showed different body composition profiles as follows: **A** a 66-year-old, non-sarcopenic woman with high-volumetric SMI; **B** a 60-year-old, sarcopenic woman with low-volumetric SMI. Red, skeletal muscle; green, abdominal visceral fat; yellow, subcutaneous fat; blue lines indicate the waist; light green line indicates L3 level. L3 level cross-sectional images are also presented

Consequently, we obtained each patient’s L3 skeletal muscle area (cm^2^) and waist skeletal muscle, visceral fat, and subcutaneous volume (cm^3^). We added visceral fat and subcutaneous fat volumes to produce total fat volume (cm^3^). The L3 skeletal muscle area was normalised to height in m^2^ and reported as the L3 skeletal muscle index (SMI), and waist volume was normalised to the height in m^3^ and reported as a volumetric index. We also calculated other body composition indices and the skeletal muscle-to-visceral fat ratio (Fig. 1).

Up to our knowledge, no studies have validated the cut-off value of L3 SMI for sarcopenia in healthy Korean women. Moreover, it was inappropriate to adopt cut-off values from previous studies conducted in other countries, as body composition varies among geographic regions and ethnicity. Instead, we defined sarcopenia when an individual’s L3 SMI was less than 39.0 cm^2^/m^2^, per the cut-off value proposed by an international consensus [22]. This cut-off value was also used in our previous studies on ovarian cancer [23] and cervical cancer [24]. Because there is no established cut-off value of the volumetric index, we used the median value of the volumetric index for each body composition component and divided patients into two groups accordingly. Thereafter, the relationships between sarcopenia, volumetric indices, and survival outcomes were evaluated.

### Statistical analysis

Baseline clinicopathologic characteristics and survival outcomes were compared between the sarcopenia and non-sarcopenia groups and between the low- and high-volumetric SMI groups. Categorical variables were compared using the Pearson’s Chi-squared test or Fisher’s exact test, while continuous variables were compared using the Student’s *t* test or Mann–Whitney *U* test. The Pearson’s correlation coefficient test was used to measure the relationship between continuous variables. For survival analyses, we used the Kaplan–Meier method with a log-rank test. Cox proportional hazards regression models were used for multivariate analyses, and adjusted hazard ratios (aHRs) and 95% confidence intervals (CIs) were calculated for each variable. All statistical analyses were conducted using the SPSS software version 25.0 (IBM Corp., Armonk, NY, USA) and GraphPad Prism 5 software (GraphPad Inc., La Jolla, CA, USA). Statistical significance was set at *p* < 0.05.

## Results

### Characteristics of the study population

Additional file 1: Figure S1 depicts the selection of the study population. In total, 385 patients were included in the analysis. The patient clinicopathologic characteristics are presented in Additional file 1: Table S1. The mean patient age was 55.5 years. Based on BMI, the proportions of overweight and obese patients were 22.6% and 43.1%, respectively. The endometrioid type was the most common histologic type (81.8%), and high-grade disease was identified in 27.5% of the study population. Two-thirds (76.6%) of the patients had early stage disease (2009 FIGO stage I–II). Pelvic lymphadenectomy and para-aortic lymphadenectomy were performed in 97.7% and 74.0% of the patients, respectively. Myometrial invasion ≥ 50% and LVSI were identified in 28.8% and 29.1% of the patients, respectively. Table 1 shows the baseline body composition of all patients. The median values for L3 SMI and volumetric SMI were 39.8 cm^2^/m^2^ (interquartile range [IQR], 33.8–46.6) and 206.0 cm^3^/m^3^ (IQR, 179.9–240.3), respectively.

**Table 1:** Baseline body composition of all patients

Characteristics	Median (IQR)
L3 sectional body composition	
*Measured*	
Skeletal muscle area, cm^2^	97.9 (82.6–114.4)
*Calculated*	
Skeletal muscle index, cm^2^/m^2^	39.8 (33.8–46.6)
Volumetric body composition	
*Measured*	
Skeletal muscle volume, cm^3^	806.9 (695.7–920.3)
Total fat volume, cm^3^	2018.9 (1484.0–2712.2)
Visceral fat volume	683.6 (399.3–1001.0)
Subcutaneous fat volume	1322.1 (1042.1–1807.3)
*Calculated*	
Skeletal muscle index, cm^3^/m^3^	206.0 (179.9–240.3)
Total fat index, cm^3^/m^3^	540.1 (388.8–711.6)
Visceral fat index, cm^3^/m^3^	177.5 (100.9–259.7)
Subcutaneous fat index, cm^3^/m^3^	351.0 (267.0–461.7)
Skeletal muscle-to-visceral fat ratio	1.153 (0.859–1.868)

Abbreviations: IQR, interquartile range

**Fig. 2:**
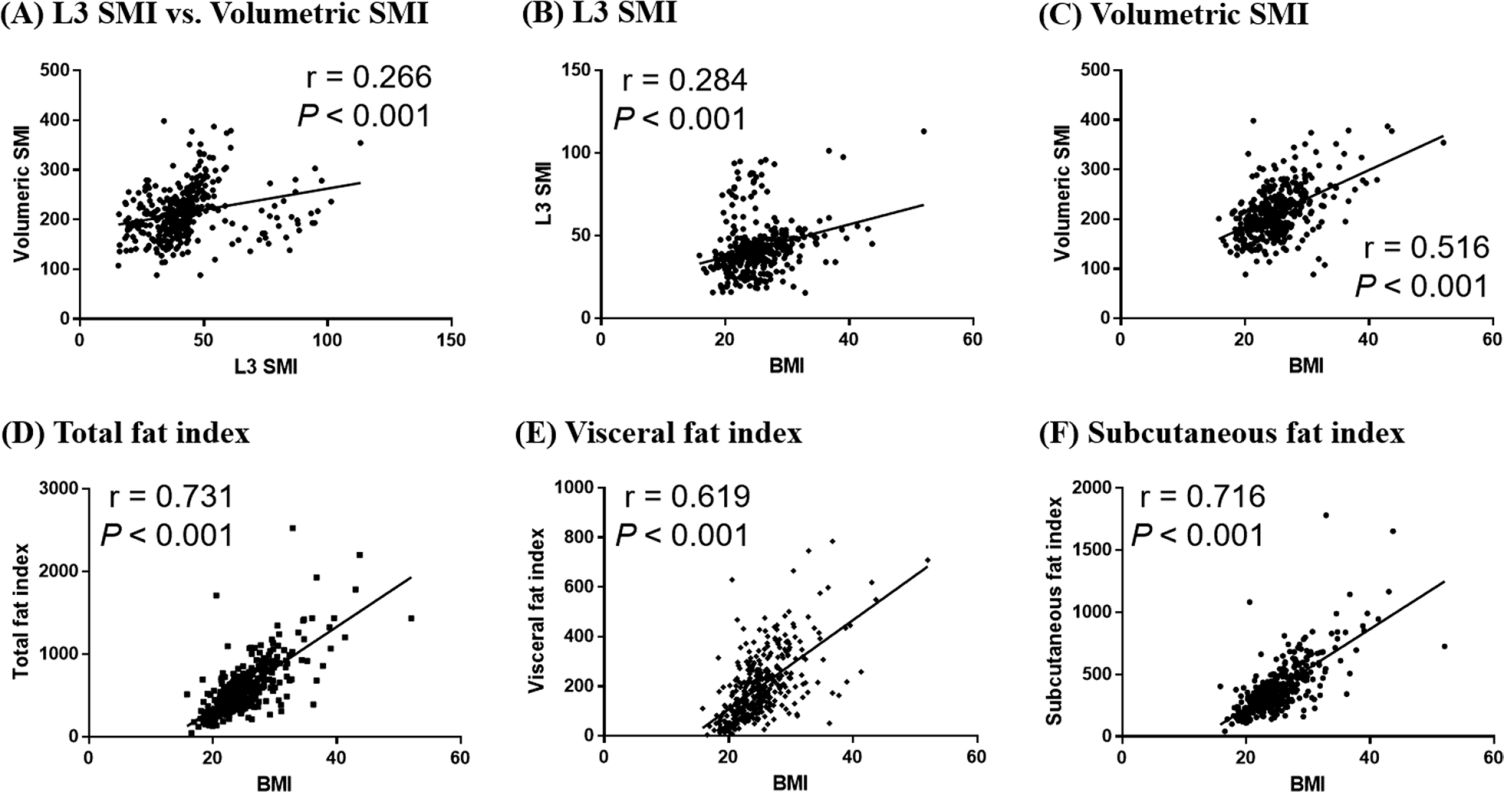
Correlations between BMI and L3 SMI and volumetric body composition indices. **A** Relationship between L3 SMI and volumetric SMI; **B** relationships between BMI and L3 SMI; **C** volumetric SMI; **D** total fat index; **E** visceral fat index; **F** subcutaneous fat index

The patients’ L3 SMI was significantly correlated with the volumetric SMI, but the relationship was weak (Pearson’s correlation coefficient *r* = 0.266; *p* < 0.001) (Fig. 2A). A significant positive correlation was observed between BMI and L3 SMI (*r* = 0.284; *p* < 0.001) and between BMI and volumetric SMI (*r* = 0.516; *p* < 0.001) (Fig. 2B, C). BMI was also correlated with volumetric total fat, visceral fat, and subcutaneous fat indices (Fig. 2D–F).

### Survival outcomes according to the various body composition indices

**Fig. 3:**
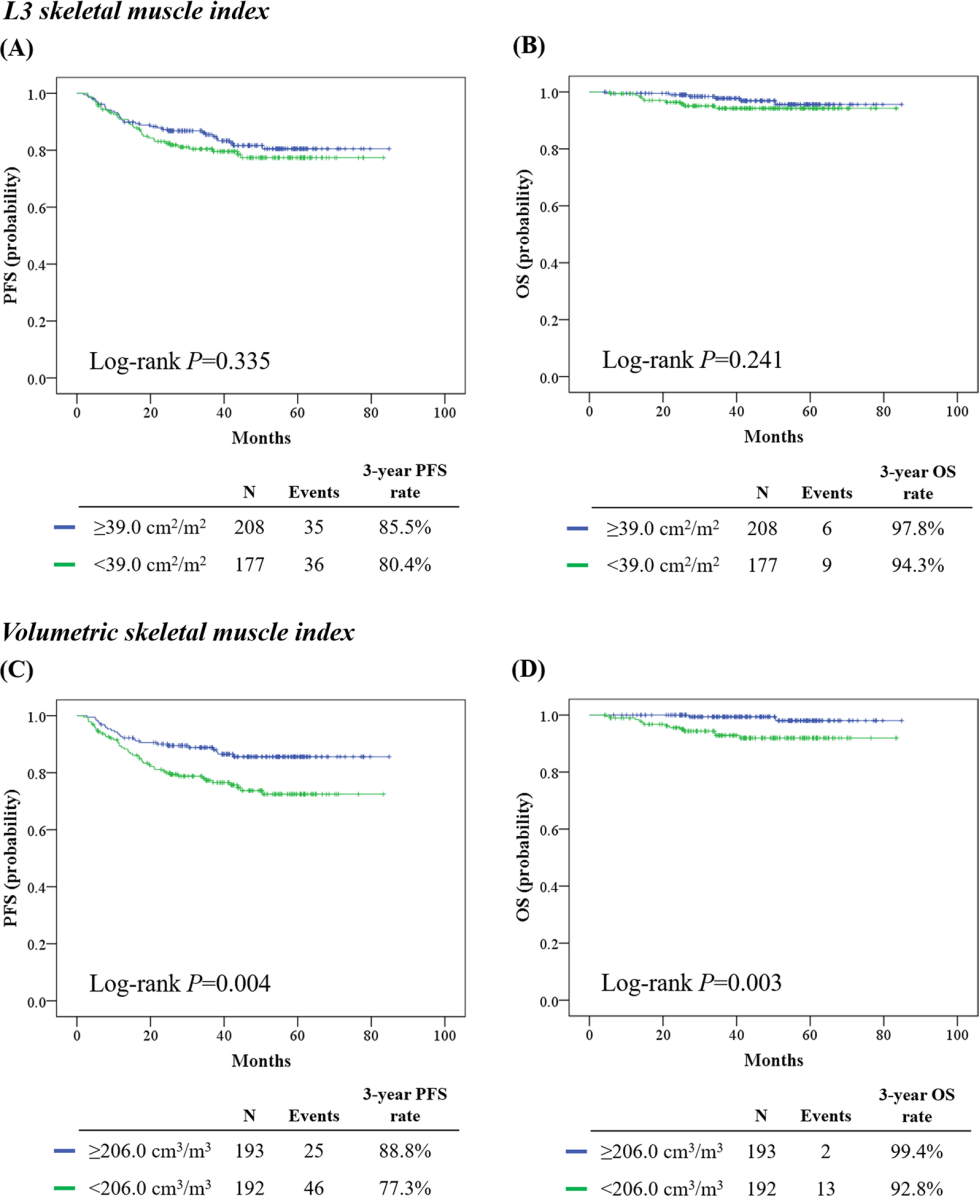
Comparisons of survival outcomes between the sarcopenia and non-sarcopenia groups (upper) and high- and low-volumetric SMI groups (lower). **A**, **C** Progression-free survival; **B**, **D** overall survival

Of the 385 patients, 71 (18.4%) experienced disease recurrence, and 15 (3.9%) died during a median observation period of 42.7 months. Based on the L3 SMI, the sarcopenia group (< 39.0 cm^2^/m^2^; *n* = 177) and non-sarcopenia group (≥ 39.0 cm^2^/m^2^; *n* = 208) showed similar PFS (3-year PFS rate, 80.4% vs. 85.5%; *p* = 0.335) and OS (3-year OS rate, 94.3% vs. 97.8%; *p* = 0.241) (Fig. 3A, B). In contrast, the low-volumetric SMI group (< 206.0 cm^3^/m^3^; *n* = 192) showed significantly worse PFS (3-year PFS rate, 77.3% vs. 88.8%; *p* = 0.004) and OS (3-year OS rate, 92.8% vs. 99.4%; *p* = 0.003), compared to the high-volumetric SMI group (≥ 206.0 cm^3^/m^3^; *n* = 193) (Fig. 3C, D). Divided by each median value, no differences in PFS and OS were observed according to the volumetric total fat, visceral fat, and subcutaneous fat indices and skeletal muscle-to-visceral fat ratio (Additional file 1: Fig. S2).

### Analyses between high- and low-volumetric SMI groups

Comparisons of clinicopathologic characteristics between the high- and low-volumetric SMI groups are presented in Table 2. Patients in the low-volumetric SMI group were significantly older (mean, 58.0 vs. 53.1 years; *p* < 0.001) and had significantly lower baseline BMI (median, 22.9 vs. 26.0 kg/m^2^; *p* < 0.001) than those in the high-volumetric SMI group. The proportion of patients with high-grade disease was significantly higher in the low-volumetric SMI group (34.9% vs. 20.2%; *p* = 0.001). Other characteristics, including comorbidities, serum CA-125 levels, histologic type, 2009 FIGO stage, pathologic risk factors, and adjuvant treatment, were similar between both groups.

**Table 2:** Clinicopathologic characteristics of high- and low-volumetric SMI groups

Characteristics	High-volumetric SMI (*n* = 193, %)	Low-volumetric SMI (*n* = 192, %)	*P*
Age, years			
Mean ± SD	53.1 ± 10.7	58.0 ± 10.6	< 0.001
BMI, kg/m^2^			
Median (IQR)	26.0 (23.7–29.1)	22.9 (21.0–25.0)	< 0.001
Underweight (< 18.5)	1 (0.5)	8 (4.2)	< 0.001
Normal (18.5–22.9)	36 (18.7)	87 (45.3)	
Overweight (23.0–24.9)	47 (24.4)	40 (20.8)	
Obesity (≥ 25.0)	109 (56.5)	57 (29.7)	
Comorbidities			
Hypertension	56 (29.0)	51 (26.6)	0.591
Diabetes	26 (31.5)	20 (10.4)	0.355
Dyslipidemia	41 (21.2)	36 (18.8)	0.541
Histologic type			0.139
Endometrioid	168 (87.0)	147 (76.6)	
Mucinous	0	1 (0.5)	
Serous	10 (5.2)	15 (7.8)	
Clear cell	2 (1.0)	6 (3.1)	
Mixed	3 (1.6)	4 (2.1)	
Carcinosarcoma	10 (5.2)	19 (9.9)	
Grade			0.005
1	103 (53.4)	82 (42.7)	
2	51 (26.4)	43 (22.4)	
3	39 (20.2)	67 (34.9)	
2009 FIGO stage			0.305
* Four categories*			
I	145 (75.1)	133 (69.3)	
II	6 (3.1)	11 (5.7)	
III	34 (17.6)	34 (17.7)	
IV	8 (4.1)	14 (7.3)	
* Two categories*			0.453
I–II	151 (78.2)	144 (75.0)	
III–IV	42 (21.8)	48 (25.0)	
CA-125, IU/ml			
Median (IQR)	18.3 (11.9–34.3)	17.3 (9.5–31.2)	0.158
Pathologic risk factors			
Myometrial invasion, ≥ 50%	49 (25.4)	62 (32.3)	0.135
LVSI	52 (26.9)	60 (31.3)	0.352
Pelvic LN metastasis^*^	26 (13.5)	26 (13.5)	0.934
Para-aortic LN metastasis^†^	13 (6.7)	11 (5.7)	0.657
Adjuvant treatment			0.681
No	118 (61.1)	111 (57.8)	
Radiation only	22 (11.4)	23 (12.0)	
Chemotherapy only	25 (13.0)	33 (17.2)	
CCRT	28 (14.5)	25 (13.0)	

Abbreviations: BMI, body mass index; CA-125, cancer antigen 125; CCRT, concurrent chemoradiation therapy; FIGO, International Federation of Gynecology and Obstetrics; IQR, interquartile range; LN, lymph node; LVSI, lymphovascular space invasion; SD, standard deviation; SMI, skeletal muscle index

Not performed: ^*^9; ^†^100

The baseline body composition of the two groups is presented in Additional file 1: Table S2. The low-volumetric SMI group had significantly lower L3 SMI (median, 33.8 vs. 44.4 cm^2^/m^2^; *p* < 0.001) and a higher proportion of L3 SMI-determined sarcopenia (64.1% vs. 28.0%; *p* < 0.001) than the high-volumetric SMI group. Volumetric total fat, visceral fat, and subcutaneous fat indices were also lower in the low-volumetric SMI group. However, the skeletal muscle-to-visceral fat ratio was higher in the low-volumetric SMI group (1.340 vs. 1.046; *p* < 0.001) than in the high-volumetric SMI group (Additional file 1: Table S2).

Associations between clinicopathologic characteristics and volumetric SMI were investigated. As shown in Additional file 1: Table S3, patients aged ≥ 55 years (median, 196.6 vs. 217.3 cm^3^/m^3^; *p* < 0.001) and patients included in underweight–normal BMI categories (median, 189.4 vs. 222.7 cm^3^/m^3^; *p* < 0.001) had significantly lower volumetric SMI, compared to those aged < 55 years and those included in overweight–obese BMI categories, respectively. However, 2009 FIGO stage was not associated with baseline volumetric SMI (four categories, *p* = 0.608; and 2009 FIGO stage I–II vs. III–IV, *p* = 0.359).

Next, we conducted multivariate analyses adjusting for patient age, FIGO stage, and other clinicopathologic factors. While BMI category was not associated with survival outcomes, low-volumetric SMI was identified as an independent poor prognostic factor for PFS (aHR, 1.762; 95% CI, 1.051–2.953; *p* = 0.032) and OS (aHR, 5.964; 95% CI, 1.296–27.448; *p* = 0.022) (Table 3 and Additional file 1: Table S4).

**Table 3:** Factors associated with patients’ progression-free survival

Characteristics	*N*	Univariate analysis			Multivariate analysis		
		HR	95% CI	*P*	aHR	95% CI	*P*
Age, years							
< 55	175	1	–	–	1	–	–
≥ 55	210	2.338	1.394–3.921	0.001	1.916	1.108–3.312	0.020
Histologic type							
Endometrioid	315	1	–	–	1	–	–
Non-endometrioid	70	5.291	3.300–8.484	< 0.001	1.572	0.794–3.111	0.194
Grade							
Low-grade	279	1	–	–	1	–	–
High-grade	106	5.582	3.461–9.003	< 0.001	1.876	0.926–3.800	0.081
FIGO stage							
I-II	295	1	–	–	1	–	–
III-IV	90	5.291	3.308–8.464	< 0.001	2.247	1.273–3.965	0.005
Adjuvant treatment							
No	229	1	–	–	1	–	–
Yes	156	6.929	3.915–12.263	< 0.001	2.888	1.468–5.683	0.002
BMI, kg/m^2^							
Underweight to normal (< 23.0)	132	1	–	–	1	–	–
Overweight (23.0–24.9)	87	0.422	0.202–0.885	0.022	0.570	0.271–1.198	0.138
Obesity (≥ 25.0)	166	0.738	0.449–1.215	0.233	1.127	0.670–1.897	0.651
Volumetric SMI							
High	193	1	–	–	1	–	–
Low	192	2.004	1.231–3.261	0.005	1.762	1.051–2.953	0.032

Abbreviations: aHR, adjusted hazard ratio; BMI, body mass index; CI, confidence interval; FIGO, International Federation of Gynecology and Obstetrics; HR, hazard ratio; SMI, skeletal muscle index

## Discussion

In this single-institution, retrospective cohort study, we demonstrated the impact of pre-treatment sarcopenia and waist body composition on survival outcomes in patients with endometrial cancer. While CT-determined sarcopenia, defined as L3 SMI < 39.0 cm^2^/m^2^, did not affect patients’ disease recurrence and mortality rates, low-volumetric SMI (< 206.0 cm^3^/m^3^) was significantly associated with worse PFS and OS.

To date, poor survival outcomes from sarcopenia have been reported in various malignancies [8–11]. While the relationship between BMI and prognosis in endometrial cancer has been well studied, studies on the prognostic impact of sarcopenia are relatively limited. Moreover, the study population (e.g. geographic regions, ethnicities, and disease setting) and definition of sarcopenia differed among the studies, which makes interpretation difficult, apart from the conflicting results.

For example, Kuroki et al. defined sarcopenia based on the median value (4.33 cm^2^) of average psoas muscle area, measured from L3 level cross-sectional images of the pre-treatment CT scans. In that study, sarcopenia was identified as an independent poor prognostic factor for PFS (aHR, 3.99; 95% CI, 1.42–11.3), but not for OS [12]. Similar to our study, Rodrigues et al. measured L3 SMI on pre-treatment CT scans, but they used the median value (42.45 cm^2^/m^2^) to categorise patients into high- and low-L3 SMI groups. The multivariate analyses revealed that a low L3 SMI was not associated with 1-year mortality [13]. Recently, Ganju et al. reported that sarcopenia, defined as L3 SMI < 41.0 cm^2^/m^2^ from the CT scans obtained at radiation simulation, did not affect PFS and OS in patients who underwent hysterectomy and pelvic radiation [14]. While these three studies were conducted in Western populations, Lee et al.’s bi-institutional retrospective cohort study was conducted in Taiwanese population [25]. This study included 131 patients with FIGO stage III endometrial cancer who underwent staging surgery and adjuvant chemoradiotherapy. Sarcopenia was defined using L3 SMI, with a cut-off value of 39.3 cm^2^/m^2^, a value similar to that in the current study. Consistent with our study, sarcopenia was not associated with either PFS (*p* = 0.28) or OS (*p* = 0.37) [25].

To the best of our knowledge, this study is the first to adopt the concept of waist volume measurement of each body component in patients with endometrial cancer. We recognise that the L3 level image analysis is a universal and widely used method. However, the analysis of a single cross-sectional CT image at the L3 level has limitations: the distribution of abdominal muscle and fat in a single cross-sectional image might vary up to twofold and threefold, respectively, owing to the shifting of the gastrointestinal tract [18]. Such variability seemed to result in a weak relationship between the L3 SMI and volumetric SMI in the current study. Thus, the volume measurement in the waist would be more precise and reflective of the whole body composition than the areal measurement in a single cross-sectional image. In particular, the artificial intelligence-based tool executed automatic volumetric quantification of a large amount of imaging data quickly and accurately.

Unlike the L3 SMI, the volumetric SMI was significantly associated with worse survival outcomes in the current study. Compared to the L3 SMI, the low-volumetric SMI might better reflect the presence of sarcopenia. Previous studies have reported that sarcopenia is associated with increased toxicity and resistance to chemotherapy in many malignancies [8–11]. Among the many features of sarcopenia, increases in pro-inflammatory cytokines, such as IL-6 and TNF-α, may be responsible for the poor prognosis of patients with sarcopenia and endometrial cancer [26, 27]. IL-6 is known to promote tumour proliferation and resistance to chemotherapy and to trigger epithelial-to-mesenchymal transition, leading to cancer metastasis [28]. Nevertheless, some might argue that patients have already suffered cancer cachexia, presenting with sarcopenia at the time of the initial diagnosis of endometrial cancer [29]. However, 76.6% of the study population had early stage disease at the time of diagnosis, and the stage was adjusted in the multivariate analyses.

Here, we also evaluated the prognostic role of other volumetric indices. Based on Calle et al.’s large cohort study of the American population [7], we initially expected that patients with endometrial cancer who have high volumetric total fat, visceral fat, and subcutaneous fat indices, and low skeletal muscle-to-visceral fat ratio would show poor prognosis. However, none of these factors was associated with survival outcomes. These findings may originate from the obesity paradox and ethnic differences. First, researchers pointed out that tumours among obese patients have less aggressive features than those among patients with normal body weight [30]. In endometrial cancer, obese patients tend to have a good prognosis for type 1 tumours, rather than poor prognosis type 2 tumours [31]. Park et al. have reported that a high pre-treatment BMI did not affect PFS and OS in Korean women with endometrial cancer [32]. Similar results were observed in the current study. Next, Asian populations are less obese than Western populations, and generally have a higher body fat percentage than Western populations, even with the same BMI [19]. Thus, studies targeting other ethnic groups may show different results. The optimal cut-off values for the volumetric body component indices may also differ.

In line with the era of precision medicine, early identification of adverse body composition which might influence individuals’ survival outcomes has important clinical implications. Therefore, if an individual has a low-volumetric SMI at a high risk of disease recurrence and mortality, physicians may pay more attention during treatment and surveillance. Based on the assessment results, physicians may prescribe oral or intravenous nutritional support and best symptomatic care [33]. Physical exercise or training intervention may be recommended to increase skeletal muscle mass or prevent further muscle loss during treatment [34, 35]. To identify disease recurrence earlier, visit intervals and surveillance methods may be individualised.

Our study has several limitations. First, due to its retrospective nature, selection bias is the most problematic. During the pre-treatment workup for endometrial cancer, CT scans have not yet been routinely performed. From the institution’s Endometrial Cancer Cohort, approximately 20% of patients were excluded owing to this reason, suggesting potentially biased. Second, associations between sarcopenia and perioperative or treatment-related complications were not investigated. Third, we did not consider sequential changes in the body composition of each patient. Patients might experience loss of skeletal muscles or gain of abdominal visceral fat during adjuvant treatment. Further studies investigating whether such longitudinal changes worsen the survival outcomes of patients with endometrial cancer are warranted. Lastly, we only measured or quantified muscle area and volume, and not muscle quality, owing to the limitations of the imaging modality.

Despite these limitations, our study is the first to introduce artificial intelligence-based volumetric measurement of body composition in patients with endometrial cancer. Conducted in a single centre with clear inclusion and exclusion criteria, homogeneity in surgery, adjuvant treatment, perioperative care, and surveillance would be strengths of the current study.

## Conclusion

In conclusion, our study results suggest that waist volumetric SMI might be a novel prognostic biomarker in patients with endometrial cancer. Considering that CT scans are commonly obtained as part of diagnosis, routine artificial intelligence-based volumetric quantification of waist skeletal muscle appears feasible in patients with endometrial cancer.

## Supplementary Information

**Additional file 1.** **Table S1.** Clinicopathologic characteristics of all patients. **Table S2.** Baseline body composition of high- and low-volumetric SMI groups. **Table S3.** Associations between volumetric SMI and clinicopathologic characteristics. **Table S4.** Factors associated with patients’ overall survival. **Figure S1.** Flow diagram depicting the selection of the study population. **Figure S2.** Survival outcomes according to the various body composition indices. Volumetric total fat index (A, E); Volumetric visceral fat index (B, F); Volumetric subcutaneous fat index (C, G); Skeletal muscle-to-visceral fat ratio (D, H). (Upper) Progression-free survival; (Lower) Overall survival.

## Data Availability

The data presented in this study are also available on request from the corresponding author.

## Abbreviations

aHR: Adjusted hazard ratio
BMI: Body mass index
CA-125: Cancer antigen 125
CI: Confidence interval
CT: Computed tomography
FIGO: International Federation of Gynaecology and Obstetrics
L3: The third lumbar vertebra
LVSI: Lymphovascular space invasion
OS: Overall survival
PFS: Progression-free survival
SMI: Skeletal muscle index
WHO: World Health Organization
