# Supplementary material for: Prognostic role of computed tomography-based, artificial intelligence-driven waist skeletal muscle volume in uterine endometrial carcinoma
Source: Insights Imaging. 2021 Dec 20;12:192. doi: 10.1186/s13244-021-01134-y (PMC8688657; doi:10.1186/s13244-021-01134-y)
Supplement: Supplementary file 1 — Additional file 1. Table S1. Clinicopathologic characteristics of all patients. Table S2. Baseline body composition of high- and low-volumetric SMI groups. Table S3. Associations between volumetric SMI and clinicopathologic characteristics. Table S4. Factors associated with patients’ overall survival. Figure S1. Flow diagram depicting the selection of the study population. Figure S2. Survival outcomes according to the various body composition indices. Volumetric total fat index (A, E); Volumetric visceral fat index (B, F); Volumetric subcutaneous fat index (C, G); Skeletal muscle-to-visceral fat ratio (D, H). (Upper) Progression-free survival; (Lower) Overall survival. [file 13244_2021_1134_MOESM1_ESM.docx]

**ADDITIONAL FILE 1**

| **Table S1.** Clinicopathologic characteristics of all patients | |
| --- | --- |
| **Characteristics** | **All**  **(n=385, %)** |
| Age, years |  |
| Mean ± SD | 55.5 ± 10.9 |
| BMI, kg/m^2^ |  |
| Median (IQR) | 24.5 (22.0−27.0) |
| Underweight (<18.5) | 9 (2.3) |
| Normal (18.5−22.9) | 123 (31.9) |
| Overweight (23.0−24.9) | 87 (22.6) |
| Obesity (≥25.0) | 166 (43.1) |
| Comorbidities |  |
| Hypertension | 107 (27.8) |
| Diabetes | 46 (11.9) |
| Dyslipidemia | 77 (20.0) |
| Histologic type |  |
| Endometrioid | 315 (81.8) |
| Mucinous | 1 (0.3) |
| Serous | 25 (6.5) |
| Clear cell | 8 (2.1) |
| Mixed | 7 (1.8) |
| Carcinosarcoma | 29 (7.5) |
| Grade |  |
| 1 | 185 (48.1) |
| 2 | 94 (24.4) |
| 3 | 106 (27.5) |
| 2009 FIGO stage |  |
| I | 278 (72.2) |
| II | 17 (4.4) |
| III | 68 (17.7) |
| IV | 22 (5.7) |
| CA-125, IU/ml |  |
| Median (IQR) | 17.9 (10.8−31.7) |
| Pathologic risk factors |  |
| Myometrial invasion, ≥50% | 111 (28.8) |
| LVSI | 112 (29.1) |
| Pelvic LN metastasis^*^ | 52 (13.5) |
| Para-aortic LN metastasis^†^ | 24 (6.2) |
| Adjuvant treatment |  |
| No | 229 (59.5) |
| Radiation only | 45 (11.7) |
| Chemotherapy only | 58 (15.1) |
| CCRT | 53 (13.8) |
| Abbreviations: BMI, body mass index; CA-125, cancer antigen 125; CCRT, concurrent chemoradiation therapy; FIGO, International Federation of Gynecology and Obstetrics; IQR, interquartile range; LN, lymph node; LVSI, lymphovascular space invasion; SD, standard deviation.  Not performed: ^*^9; ^†^100. | |

| **Table S2.** Baseline body composition of high- and low-volumetric SMI groups | | | |
| --- | --- | --- | --- |
| **Characteristics** | **High-volumetric SMI (n=193, %)** | **Low-volumetric SMI**  **(n=192, %)** | ***P*** |
| **L3 sectional body composition** |  |  |  |
| *Measured* |  |  |  |
| Skeletal muscle area, cm^2^ | 107.0 (90.9−120.8) | 83.5 (65.4−91.5) | <0.001 |
| *Calculated* |  |  |  |
| Skeletal muscle index, cm^2^/m^2^ | 44.4 (37.9−49.3) | 33.8 (26.9−37.3) | <0.001 |
| L3 sarcopenia^*^ |  |  | <0.001 |
| No | 139 (72.0) | 69 (35.9) |  |
| Yes | 54 (28.0) | 123 (64.1) |  |
| **Volumetric body composition** |  |  |  |
| *Measured* |  |  |  |
| Skeletal muscle volume, cm^3^ | 919.8 (843.9−1065.7) | 742.9 (651.1−848.9) | <0.001 |
| Total fat volume, cm^3^ | 2513.5 (1984.5−3374.1) | 1867.9 (1318.9−2444.4) | <0.001 |
| Visceral fat volume | 905.0 (637.3−1188.8) | 557.1 (291.2−879.6) | <0.001 |
| Subcutaneous fat volume | 1605.7 (1257.5−2140.2) | 1232.5 (942.1−1603.8) | <0.001 |
| *Calculated* |  |  |  |
| Skeletal muscle index, cm^3^/m^3^ | 240.2 (222.8−267.9) | 191.0 (169.8−215.0) | <0.001 |
| Total fat index, cm^3^/m^3^ | 682.0 (547.3−854.3) | 473.5 (322.2−634.8) | <0.001 |
| Visceral fat index, cm^3^/m^3^ | 231.8 (171.5−330.6) | 136.8 (70.4−221.6) | <0.001 |
| Subcutaneous fat index, cm^3^/m^3^ | 427.1 (350.8−562.0) | 318.5 (234.5−411.0) | <0.001 |
| Skeletal muscle-to-visceral fat ratio | 1.046 (0.761−1.491) | 1.340 (0.908−2.667) | <0.001 |
| Presented as median value with interquartile range.  ^*^L3 sarcopenia was defined when L3 SMI was less than 39.0 cm^2^/m^2^. | | | |

| **Table S3.** Associations between volumetric SMI and clinicopathologic characteristics | | | |
| --- | --- | --- | --- |
| **Characteristics** | **All**  **(n=385, %)** | **Volumetric SMI**  **(median, IQR)** | ***P*** |
| Age, years |  |  | <0.001 |
| <55 | 175 (45.5) | 217.3 (188.0−264.0) |  |
| ≥55 | 210 (54.5) | 196.6 (173.8−229.7) |  |
| BMI, kg/m^2^ |  |  |  |
| *Four categories* |  |  | <0.001 |
| Underweight (<18.5) | 9 (2.3) | 179.7 (152.4−212.7) |  |
| Normal (18.5−22.9) | 123 (31.9) | 190.6 (167.5−205.3) |  |
| Overweight (23.0−24.9) | 87 (22.6) | 205.9 (184.2−235.8) |  |
| Obesity (≥25.0) | 166 (43.1) | 232.2 (194.0−265.1) |  |
| *Two categories* |  |  | <0.001 |
| Underweight−Normal (<23.0) | 132 (34.3) | 189.4 (164.0−206.7) |  |
| Overweight−Obesity (≥23.0) | 253 (65.7) | 222.7 (189.1−256.6) |  |
| L3 sarcopenia^*^ |  |  | <0.001 |
| No | 177 (46.0) | 190.9 (170.2−214.8) |  |
| Yes | 208 (54.0) | 224.2 (194.5−260.7) |  |
| 2009 FIGO stage |  |  |  |
| *Four categories* |  |  | 0.608 |
| I | 278 (72.2) | 210.0 (182.5−242.9) |  |
| II | 17 (4.4) | 199.2 (183.0−225.7) |  |
| III | 68 (17.7) | 203.2 (175.5−235.6) |  |
| IV | 22 (5.7) | 198.3 (166.1−249.7) |  |
| *Two categories* |  |  | 0.359 |
| I−II | 295 (76.6) | 208.4 (182.7−241.5) |  |
| III−IV | 90 (23.4) | 198.7 (174.9−236.2) |  |
| Abbreviations: BMI, body mass index; FIGO, International Federation of Gynecology and Obstetrics; IQR, interquartile range; SMI, skeletal muscle index.  ^*^L3 sarcopenia was defined when L3 SMI was less than 39.0 cm^2^/m^2^. | | | |

**Table S4.** Factors associated with patients’ overall survival

| **Characteristics** | ***N*** | ***Univariate analysis*** | | | ***Multivariate analysis*** | | |
| --- | --- | --- | --- | --- | --- | --- | --- |
|  |  | **HR** | **95% CI** | ***P*** | **aHR** | **95% CI** | ***P*** |
| Age, years |  |  |  |  |  |  |  |
| <55 | 175 | 1 | − | − | 1 | − | − |
| ≥55 | 210 | 3.613 | 1.019−12.804 | 0.047 | 3.397 | 0.886−13.020 | 0.074 |
| Histologic type |  |  |  |  |  |  |  |
| Endometrioid | 315 | 1 | − | − | 1 | − | − |
| Non-endometrioid | 70 | 9.533 | 3.376−26.923 | <0.001 | 2.497 | 0.517−12.065 | 0.255 |
| Grade |  |  |  |  |  |  |  |
| Low-grade | 279 | 1 | − | − | 1 | − | − |
| High-grade | 106 | 9.224 | 2.931−29.032 | <0.001 | 1.697 | 0.295−9.754 | 0.553 |
| FIGO stage |  |  |  |  |  |  |  |
| I-II | 295 | 1 | − | − | 1 | − | − |
| III-IV | 90 | 14.898 | 4.202−52.822 | <0.001 | 8.130 | 1.817−36.388 | 0.006 |
| Adjuvant treatment |  |  |  |  |  |  |  |
| No | 229 | 1 | − | − | 1 | − | − |
| Yes | 156 | 11.003 | 2.481−48.802 | 0.002 | 1.948 | 0.349−10.878 | 0.447 |
| BMI, kg/m^2^ |  |  |  |  |  |  |  |
| Underweight to normal (<23.0) | 132 | 1 | − | − | 1 | − | − |
| Overweight (23.0−24.9) | 87 | 0.666 | 0.172−2.576 | 0.556 | 1.256 | 0.308−5.126 | 0.751 |
| Obesity (≥25.0) | 166 | 0.566 | 0.180−1.784 | 0.331 | 0.988 | 0.304−3.210 | 0.984 |
| Volumetric SMI |  |  |  |  |  |  |  |
| High | 193 | 1 | − | − | 1 | − | − |
| Low | 192 | 7.014 | 1.583−31.086 | 0.010 | 5.964 | 1.296−27.448 | 0.022 |
| Abbreviations: aHR, adjusted hazard ratio; BMI, body mass index; CI, confidence interval; FIGO, International Federation of Gynecology and Obstetrics; HR, hazard ratio; SMI, skeletal muscle index. | | | | | | | |

**Fig. S1.** Flow diagram depicting the selection of the study population.


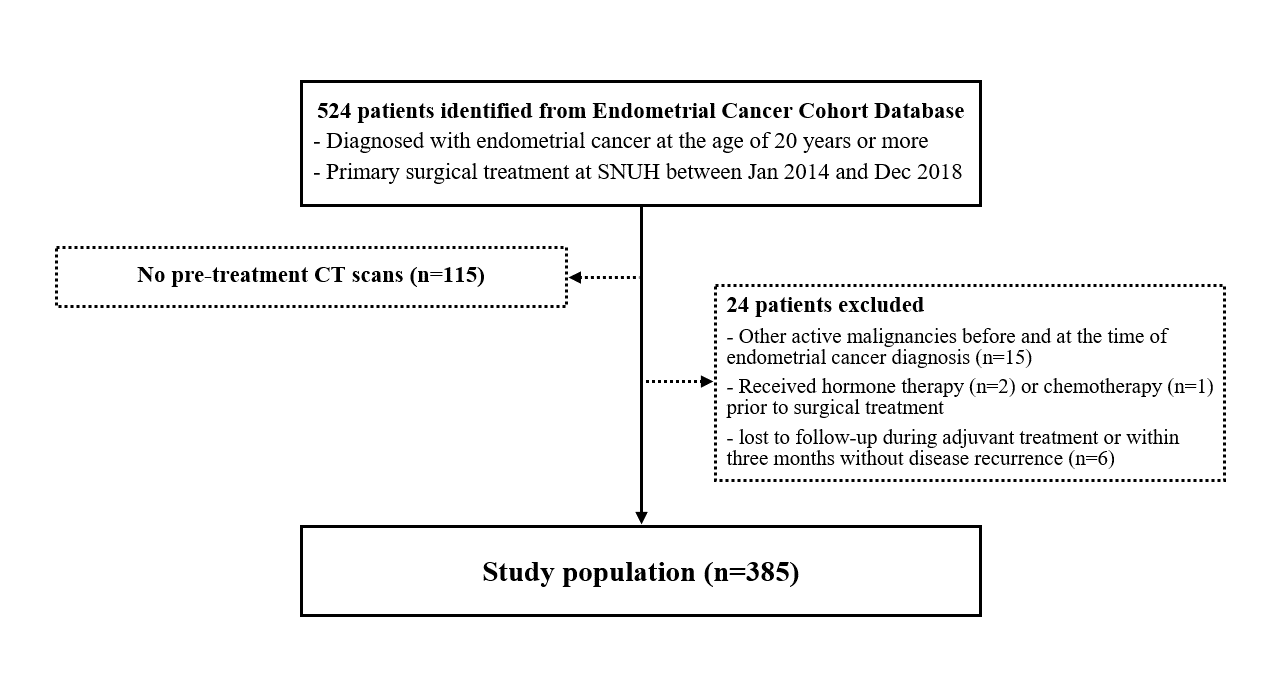


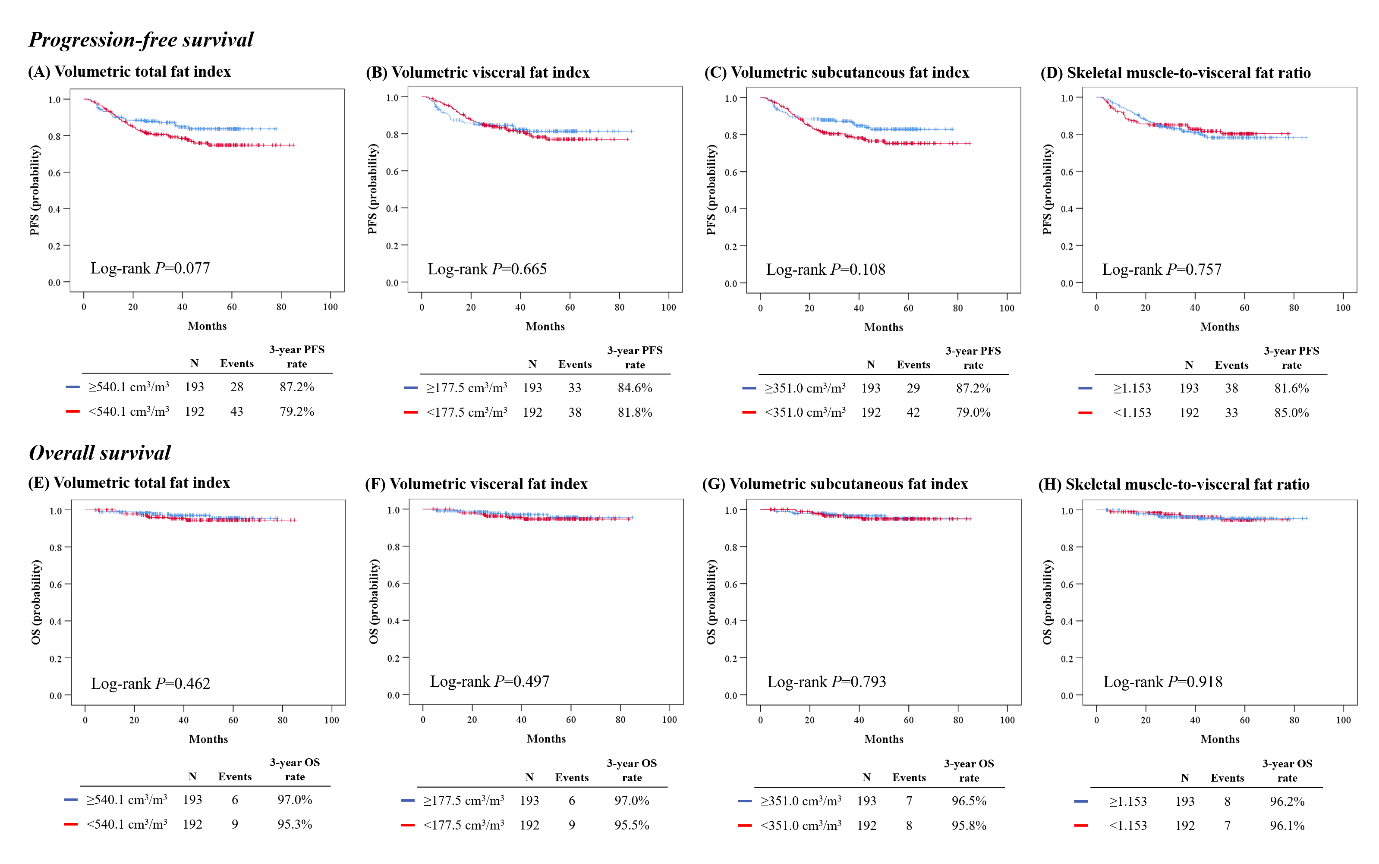
**Fig. S2.** Survival outcomes according to the various body composition indices. Volumetric total fat index (A, E); Volumetric visceral fat index (B, F); Volumetric subcutaneous fat index (C, G); Skeletal muscle-to-visceral fat ratio (D, H). (Upper) Progression-free survival; (Lower) Overall survival.
